# A Biophysical Model of Cell Adhesion Mediated by Immunoadhesin Drugs and Antibodies

**DOI:** 10.1371/journal.pone.0019701

**Published:** 2011-05-18

**Authors:** Ryan N. Gutenkunst, Daniel Coombs, Toby Starr, Michael L. Dustin, Byron Goldstein

**Affiliations:** 1 Department of Molecular and Cellular Biology, University of Arizona, Tucson, Arizona, United States of America; 2 Department of Mathematics and Institute of Applied Mathematics, University of British Columbia, Vancouver, British Columbia, Canada; 3 Department of Pathology, New York University School of Medicine and Program in Molecular Pathogenesis, Skirball Institute of Biomolecular Medicine, New York, New York, United States of America; 4 Theoretical Biology and Biophysics Group, Theoretical Division, Los Alamos National Laboratory, Los Alamos, New Mexico, United States of America; Université Paris Descartes, France

## Abstract

A promising direction in drug development is to exploit the ability of natural killer cells to kill antibody-labeled target cells. Monoclonal antibodies and drugs designed to elicit this effect typically bind cell-surface epitopes that are overexpressed on target cells but also present on other cells. Thus it is important to understand adhesion of cells by antibodies and similar molecules. We present an equilibrium model of such adhesion, incorporating heterogeneity in target cell epitope density, nonspecific adhesion forces, and epitope immobility. We compare with experiments on the adhesion of Jurkat T cells to bilayers containing the relevant natural killer cell receptor, with adhesion mediated by the drug alefacept. We show that a model in which all target cell epitopes are mobile and available is inconsistent with the data, suggesting that more complex mechanisms are at work. We hypothesize that the immobile epitope fraction may change with cell adhesion, and we find that such a model is more consistent with the data, although discrepancies remain. We also quantitatively describe the parameter space in which binding occurs. Our model elaborates substantially on previous work, and our results offer guidance for the refinement of therapeutic immunoadhesins. Furthermore, our comparison with data from Jurkat T cells also points toward mechanisms relating epitope immobility to cell adhesion.

## Introduction

When a pathogen elicits a humoral immune response, antibodies are produced that bind to specific epitopes on the surface of the pathogen. Once antibodies have bound to the pathogen, it is labeled as foreign, and various processes can follow that lead to its elimination. One such process, antibody-dependent cell-mediated cytotoxicity (ADCC), involves natural killer (NK) cells binding through their Fc
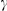
RIIIa (CD16a) receptors to IgG antibodies decorating the pathogen (reviewed in [1]). The coupling of an NK cell to a target cell brings parts of the surfaces of the two cells into proximity, within roughly 100Å. In the region of tight contact where antibodies form bridges between the two cells, both the density of epitopes on the target cell and the density of Fc receptors on the NK cell are locally increased. When the density of Fc receptors in the contact region on the NK cell is sufficiently high, a cellular response is triggered, the end point of which is the release of lytic granules containing perforin and granzymes, whose combined effect results in the killing of the target cell [2]–[4]. Depending on the nature of the epitope and type of cell, the aggregation of epitopes on the target cell may also trigger cellular responses [5], [6].

Monoclonal antibodies and antibody-like fusion proteins have been developed to take advantage of ADCC. These drugs target naturally occurring proteins that are overexpressed on tumor cells and on populations of cells that drive autoimmune responses [1], [7]–[10]. Unfortunately, these drugs will also target a subset of healthy cells because the target is a naturally occurring protein. An obvious question, which we address in this paper, is what properties of a drug, the cells that express the target protein, and the NK cells determine a drug's ability to discriminate between pathogenic and healthy cells? A second question that we consider, that is closely related to the first, is what determines the range of drug concentrations over which a drug will couple target cells to NK cells? These drugs, either in animal models or patients, must compete for Fc receptors on NK cells with endogenous IgG [11]. We therefore also examine how background IgG influences the range of drug concentrations over which adhesion occurs.

We previously presented an equilibrium model that describes the coupling via a monoclonal antibody (or an appropriate fusion protein) of identical target cells to a surface expressing mobile Fc receptors [12]. Here, we significantly extend our model to allow for a target cell population with a distribution of surface epitope density. This allows us to analyze experiments where the percentage of bound target cells is determined as a function of the ligand concentration. We also extend the model to admit the possibility of nonspecific adhesion between target cells and the surface. Our extended model also addresses the possibility that some fraction of the target epitopes are immobile, including cases in which the immobile fraction depends on epitope cross-linking or the size of the contact region. These cases model some potential target cell responses to adhesion.

To test predictions of the model, we use an experimental system consisting of a planar bilayer containing mobile Fc
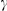
RIIIb (CD16b) receptors, Jurkat T cells expressing the cell-adhesion molecule CD2, and the drug alefacept that binds the target cell to the bilayer [12]. Fc
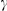
RIIIb differs from Fc
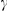
RIIIa, the receptor on NK cells, in that it lacks a transmembrane region and a cytoplasmic tail and it anchors to membranes via glycosolphosphatidylinositol [13]. Further, the extracellular domains of the two receptors differ by six amino acids, which probably accounts for Fc
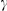
RIIIb having a lower affinity for IgG than Fc
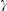
RIIIa [13], [14]. Alefacept is a recombinant fusion protein that has an antibody-like architecture where the Fab binding sites have been replaced by the natural ligand for CD2, the extracellular domain of CD58 [15], [16], and fused to the human IgG1 hinge, C
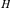
2, and C
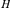
3 domains [2]. It is used in the treatment of psoriasis, an autoimmune disease. Alefacept reduces the number of circulating memory-effector T cells in treated patients and mediates ADCC in vitro [2], [17]–[20].

Alefacept is an example of an immunoadhesin, which is a molecule that uses the basic framework of an IgG antibody, but replaces the Fab binding sites with the ectodomain of an adhesion molecule. Immunoadhesins have the specificity of an adhesion molecule as well as some properties of an antibody, such as the ability to bind to Fc receptors and a long half-life in plasma that is similar to IgG [21], [22]. An interesting property of alefacept is that it mediates adhesion and killing of target cells by NK cells at nM concentrations [12], even though both the binding of IgG to Fc
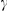
RIIIa and the binding of CD58 to CD2 [23] are low affinity, with dissociation constants in the 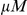 range. The model we present will show how the range of drug concentrations over which adhesion occurs depends on these equilibrium constants as well as the other parameters of the system.

## Methods

We consider a population of target cells expressing a particular epitope, with some fraction of the epitopes freely diffusing in the target cell membrane and the remainder immobile (i.e., fixed in position on the membrane). Additionally, we consider a bilayer with mobile receptors diffusing on its surface and a ligand capable of simultaneously binding both the epitope and the receptor through different sites. The ligand is either a monoclonal antibody or an immunoadhesin; its Fab arms bind monovalently or divalently to the epitope on the target cells, and its Fc leg binds monovalently to the receptor on the bilayer. At some ligand concentration a contact region forms between the cell and the bilayer; its area is an increasing function of the number of ligand-mediated bridging bonds that form. The ligand also competes for receptor binding with nonspecific antibodies that cannot form bridging bonds.

### Concentrations and equilibrium constants

**Figure 1:**
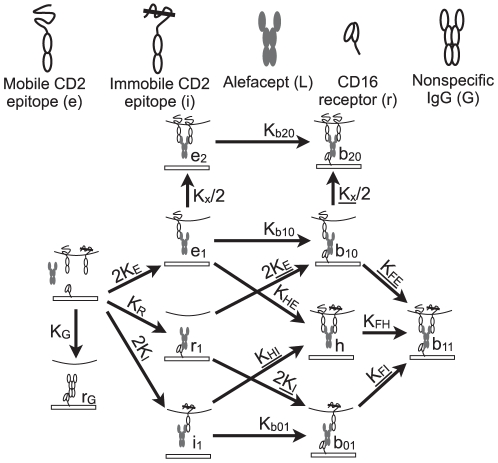
Model reaction network. All molecular species and reactions are labeled. All reactions are reversible; the arrow in the figure denotes the forward direction for defining the equilibrium constant, which labels the arrow. Underlined rate constants are eliminated using detailed balance.

The potential reactions among mobile and immobile epitopes, ligand, receptor, and nonspecific antibody are illustrated in Fig. 1. Each molecular complex is labeled by our mathematical notation for its surface concentration. All species except those involving a bridging bond (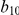, 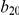, 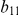, and 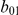) exist both inside and outside the contact region, and the subscript ‘in’ denotes species inside the contact region. Detailed balance places six constraints on the equilibrium constants, which we use to eliminate the underlined constants in Fig. 1 (Text S1). To find the equilibrium state of this system for any given bulk ligand concentration 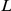, we solve five algebraic equations for five unknowns: the free immobile epitope concentrations outside 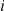 and inside 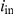 the contact region, the free mobile epitope 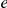 and receptor 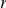 concentrations outside the contact region, and the fraction of the target cell surface 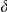 comprising the contact region. To make our analysis tractable, we make several simplifying assumptions regarding the equilibrium configuration of receptors and epitopes.

Our first assumption is that the equilibrium constants for reactions involving immobile epitopes are identical to the corresponding constants involving mobile epitopes: 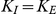, 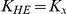, 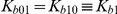, and 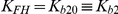. Making this assumption substantially reduces the number of unknown parameters. We expect that this assumption leads to negligible error, because the relevant physical interactions are identical for mobile and immobile epitopes. In particular, because they are equilibrium constants, these parameters are not altered by the difference in diffusivity between mobile and immobile epitopes.

Our second assumption is that the typical distance between immobile epitopes on the target cell is large compared with the span of the two arms of the ligand, so that the ligand cannot cross-link immobile epitopes. Thus we do not consider complexes containing more than one immobile receptor. Given a CD2 surface density 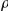, and assuming that the CD2 epitopes are uniformly distributed, the probability 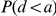 that an epitope's nearest neighbor is a distance 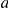 away or closer is [24]

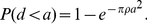
(1)As detailed later, each T-cell contains of order 64,000 CD2 epitopes, over a surface area of roughly 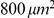, yielding a density of 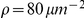. Given this density, the probability that an epitope's nearest neighbor is closer than the span of roughly 10 nm [25] between epitope binding arms of an antibody-like molecule, such as alefacept, is less than 3%. We thus expect that cross-links between immobile epitopes will indeed be rare, because the density of immobile epitopes is even lower than the total epitope surface density.

Our third assumption relates the free mobile epitope and receptor concentrations inside and outside the contact region. In earlier experiments, fluorescently labeled CD48 (Cy5-CD48) was coupled to the bilayer, and it was observed that the fluorescence from CD48 was reduced in the contact region to approximately 75% of its value outside the contact region [12]. This suggests that the contact region introduces steric hindrance and partitions mobile surface proteins between the inside and outside of the contact region. We assume that at equilibrium 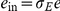 and 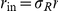, where 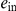 and 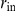 are respectively the free epitope and free receptor concentrations inside the contact region and 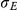 and 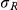 are equilibrium partition coefficients. The partition coefficients for Fc
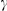
RIIIb and CD2 have not been determined; we assume they behave similarly to CD48 because they are of similar size, so we take 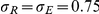. In Figure S1A we explore the sensitivity of our results to this assumption.

Using the law of mass action and these additional assumptions, we can write the equilibrium concentration of all bound complexes in terms of the free epitope, receptor, ligand, and nonspecific antibody concentrations. For example, the concentration 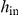 of complexes inside the contact region consisting of a ligand cross-linking a mobile and an immobile epitope is
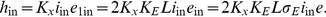
(2)The factor of 2 in calculating the concentration 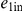 of complexes between a ligand and mobile epitope arises because 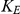 is a single-site equilibrium constant, and there are two potential binding sites on the ligand. Similarly, the concentration 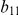 of bridging complexes involving a receptor, a mobile epitope, and an immobile epitope is
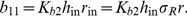
(3)The full system of equilibrium relations is given in Text S1.

Given our assumptions, in the limit that target cells are sparse, the equilibrium state will depend on six equilibrium constants (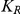, 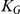, 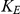, 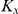, 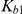, and 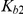); two partition coefficients (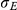 and 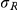); the total receptor 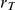, epitope 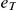, ligand 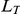, and nonspecific antibody 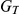 concentrations; and the epitope immobile fraction 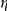. Additionally, two parameters 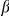 and 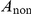 (detailed later) relate the area of the contact region to the number of bridging bonds. Finally, to connect our model with the data, we require the surface area 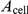 of the Jurkat T cells studied. In our analyses, a number of these parameters were held fixed (Table 1).

**Table 1:** Fixed parameter values.

parameter	value	reference
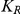	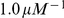	[35]–[37]
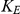	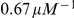	[16]
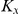	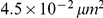	this work
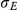	0.75	[12]
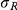	0.75	[12]
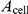	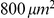	[12]

Most of our fixed parameter values come directly from measurements; an exception is 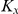, the cross-linking constant for alefacept binding. To estimate 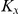, we equate the measured apparent dissociation constant 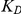 for alefacept adhering to T cells to the inverse of the initial slope 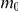 of a Scatchard plot for a bivalent ligand binding to a monovalent receptor [26]:
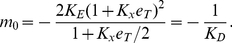
(4)Using the mean CD2 count measured for our cells of 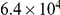 and 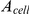 to calculate 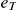, along with 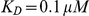 [12] yields the value for 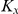 in Table 1. With the exception of the fit parameter 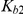, our results are insensitive to the precise value of 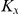 (Figure S1B).

### Conservation laws

In the experiments we consider, there is negligible depletion of ligand, so the free ligand concentration is well approximated by the total ligand concentration (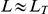). Similarly, in the experiments with nonspecific antibody, the antibody is negligibly depleted, so 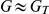. Conservation of epitopes and receptors, however, introduces additional constraints on the concentrations of various complexes.

In our model, there are three classes of epitopes: mobile epitopes, immobile epitopes outside the contact region, and immobile epitopes inside the contact region. We assume that the concentrations of all species have reached equilibrium. For mobile epitopes, we have
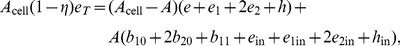
(5)where 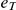 is the average epitope density on the cell surface, equal to the total epitope count 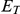 divided by the cell area 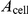, and 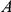 is the area of the contact region. This equation expresses the fact that the total number of mobile epitopes (left-hand side) must be equal to the total number in complexes outside and inside the contact region (right-hand side). In terms of the fraction 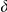 of the cell surface in the contact region, the above conservation law is
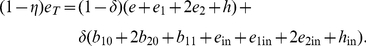
(6)Similarly, for immobile epitopes outside the contact region we have
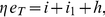
(7)and for immobile epitopes inside the contact region we have
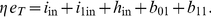
(8)


In our analyses, we consider fits with no immobile epitopes, a constant immobile fraction 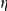, and a varying immobile fraction 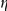 that is a function of either 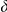 or the fraction 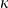 of target cell epitopes that are cross-linked by ligand:
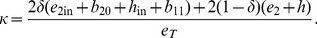
(9)For the varying 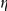 cases, we consider a linear dependence of 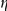 on 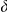 or 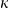:
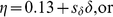
(10)

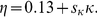
(11)Here 0.13 is the experimentally observed immobile epitope fraction in the absence of ligand [12].

For receptors in the bilayer, we have
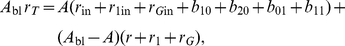
(12)where 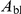 is the total area of the bilayer divided by the number of adhered cells. Dividing by 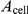 and rearranging yields
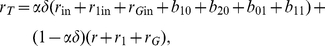
(13)where 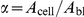. In the experiments we analyze, for all ligand concentrations the adhered cells are sparsely distributed over the bilayer, so we take 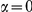. In this case, the free receptor concentration 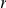 is simply
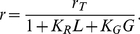
(14)


### Contact region growth law

Bell, Dembo, and Bongrand argued [27] that the bridging bond density between two adhered cells is determined by a constant repulsive pressure arising from electrostatic repulsion caused by negative charges associated with cell surfaces and steric stabilization effects. The steric effects arise because cell membranes are coated by a hydrated layer of long-chain polymers (glycocalyces) that must compress as cells are brought together and water is squeezed out of the contact region. Together with the assumption that cells are easily deformed, this argument implies that the area of the contact region grows linearly with the number of bridging bonds.

Although we expect the repulsion between our target cells and bilayer to be smaller than that between two cells, we expect the repulsive forces to be of similar origin. Moreover, in our experiments the small contact regions observed cause only small cellular deformations, and our target Jurkat T cells are substantially easier to deform than some other cell types, such as neutrophils and HL60 cells [28]. However, a recent study of the binding of Jurkat T cells to bilayers containing the natural CD2 ligand CD58 observed that the average contact area did not go to zero as the average number of bridging bonds went to zero [29], and a similar effect may be present in our data. To allow for this possibility, we include a nonspecific adhesion area 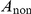 into our contact area growth law, so that the total contact area 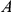 is equal to the the nonspecific contribution 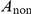 plus a specific contribution proportional to the total number of bridging bonds:
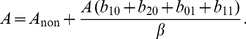
(15)Dividing both sides by 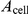, we obtain
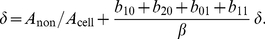
(16)The parameter 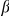 is the bridging bond density required to balance the repulsive force per unit area between the cell and the bilayer.

Solving the five constraint equations (Eq. 6, 7, 8, 13, and 16) for the five unknowns (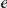, 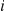, 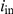, 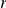, and 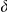) allows us to calculate the area of the contact region for a cell with a specified epitope density 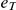 given the ligand concentration 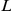.

For physical (i.e., non-negative) concentrations of bridging bonds, Eq. 16 strictly constrains the contact area 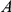 to be greater than the nonspecific contact area 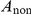. In our data, we observe that not all cells adhere at low and high ligand concentrations, corresponding to some cells with 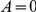. This suggests that nonspecific forces do not help cells initiate adhesion, but rather act to increase the contact area after specific drug-mediated interactions have created contact. Thus when calculating whether or not a cell with a specific epitope density will adhere, we take 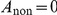. Then, for a fixed epitope density, adhesion occurs over a range of soluble ligand concentrations: 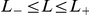. Similarly, for a fixed ligand concentration, adhesion occurs only above a minimal epitope density 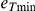. We calculate this minimal density by solving for 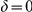 with 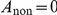. We expect that taking 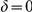 rather than setting it equal to some minimal value introduces negligible error in our estimates of 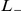, 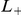, and 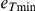. In our experiments adhesion was determined in the absence of flow, based on whether there was accumulation of the receptor CD58 in the contact area. Even in the presence of weak flows, estimates suggest that few bonds, and thus small contact areas, are needed to initiate adhesion [30], [31].

We solve the constraint equations and perform all numerics using the Python library SciPy [32]. Uncertainties on fit parameters are calculated via bootstrapping, with 100 bootstrap data sets for each model.

### Heterogeneous density of epitopes on target cells

We now consider a target cell population with a normalized distribution 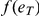 of epitope densities. For a given ligand concentration, if 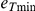 is the minimum epitope density at which adhesion will occur, the fraction of cells bound to the bilayer is
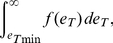
(17)and the average area of the contact region is
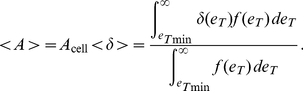
(18)


We would like to use a continuous distribution 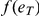 in our model that is flexible enough to approximate a wide range of epitope distributions and is relatively simple to work with. Of the few well-known continuous distributions where the variable 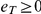, the Weibull distribution fits our needs. The Weibull distribution has density
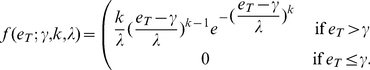
(19)In our fits, we fix the location parameter 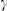 to zero. The parameter 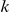 is the Weibull shape parameter. For 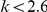, 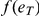 is skewed to the right, for 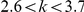 it is essentially unskewed and looks like a normal distribution, and for 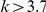 it is skewed to the left. For 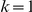 and 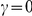, the Weibull distribution reduces to the exponential distribution. The mean of the Weibull distribution is given by 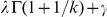, where 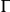 denotes the Gamma function. We report 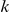 and mean total epitope count 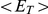, because the total epitope count is most directly connected with experimental measurements. From 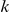 and 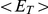, 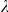 can be calculated by inverting the formula for the mean of the Weibull distribution and dividing by 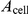.

**Figure 2:**
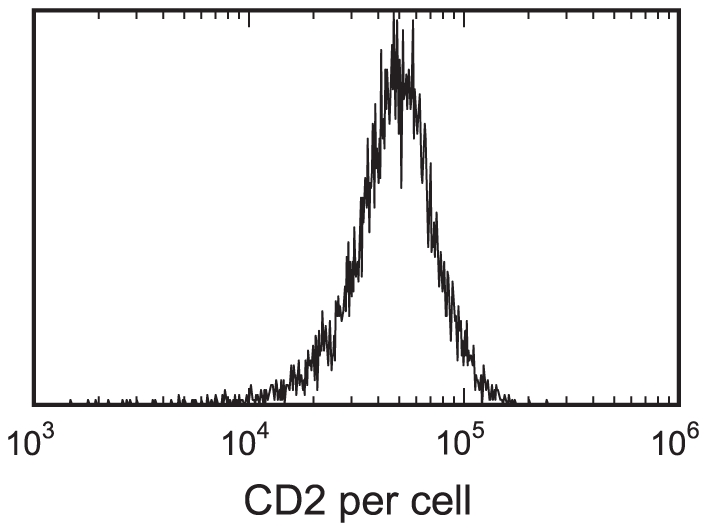
Distribution of CD2 epitope count on Jurkat T cells. Values were determined by flow cytometry.

Fig. 2 shows the distribution of expression of CD2 on the surfaces of a population of Jurkat T cells (the target cells in our study) as determined by flow cytometry. This distribution serves as a baseline to judge the distributions arising from our model fits. These measurements were performed on a Becton Dickinson FacsCaliber. Fluorescein isothiocyanate (FITC) labeling of antibodies and the determination of the fluoresceine∶protein ratio was determined using absorption spectroscopy [33]. Calibration was performed using FITC standard beads obtained from Bangs Laboratories (Fishers, IN). Antibody-stained cells were washed twice prior to analysis.

### Numerical solution

In general, calculating 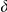 involves numerically solving a system of five algebraic equations (Eq. 6, 7, 8, 13, and 16). To find the the bounding ligand (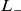 and 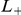) and epitope (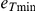) concentrations for adhesion, we employ numerical root-finding for 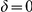 with 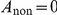, and we calculate 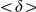 by explicit numerical integration of 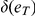 over the distribution 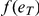 from 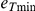 to infinity. In some special cases, additional simplifications are possible that dramatically reduce computational difficulty.

When all epitopes are mobile (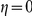), there is no depletion of bilayer receptors (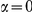), and there are no nonspecific forces (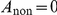), the model simplifies substantially to a system of three equations for 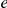, 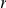, and 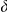. In this case, we can derive polynomial equations for the bounding ligand concentrations 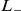 and 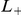 and epitope density 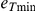 for adhesion. Setting 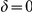 in Eq. 6, 13, and 16 and substituting our expressions for equilibrium species concentrations (such as Eq. 2) yields a system of three equations for the unknowns 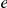, 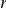, and 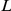. For a fixed epitope density 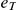, this system can be reduced to a single cubic equation in 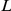, which is given in Text S1. This cubic equation may have either two positive roots (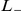 and 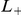) or no positive roots (no adhesion irrespective of 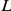). Similarly, for a fixed ligand concentration 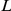, the system of three equations can be reduced to a quadratic equation for 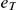, the larger root of which is 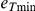 (Text S1). Additionally, in this special case, we can show that 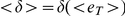, where 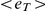 is the average epitope density of adhered cells (Text S1). This saves us from having to numerically integrate over 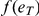.

When 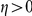, direct solution of our system of five constraint equations will give non-physical results for those cases in which the immobile epitopes themselves are dense enough to drive adhesion. Because the immobile epitope density is assumed constant over the cell surface, this case results in a divergent contact area 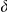, as demonstrated in Figure S2. In our application, this phenomenon only occurs at very high total epitope densities 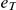, so it makes only a small contribution in our typical integrations over 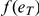, but we must handle it carefully to avoid numerical difficulties. When the contact area is divergent, Eq. 16 implies that the total concentration of bridging bonds must be equal to 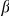. Because it is the immobile epitopes that are causing this divergence, the bridging bonds are dominated by 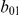; therefore, the value of 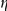 which leads to this divergence, 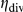, can be found by solving our conservation equation for immobile epitopes inside the contact region (Eq. 8) with 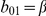, yielding
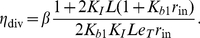
(20)When 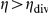, we set 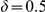, consistent with a cell completely flattened against the surface. Similarly, we set 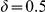 whenever direct solution of the equations would yield a larger value for 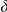. Altering this maximum value of 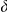 has minor influence on our results, because in our experiments the vast majority of cells have only a small fraction of their area adhered.

When we consider the immobile fraction to be a function of crosslinked epitopes or of the contact area, then Eq. 10 or 11 represents an additional constraint to our previous five, to account for the additional free variable 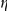. There may be multiple self-consistent solutions of our expanded system of six constraint equations (Figure S2). Physically, we expect the cell to adopt the solution corresponding to smaller 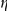, as the cell begins in a state with minimal epitope immobility. In our calculations we always adopt the smallest possible solution, by beginning our root-finding from small 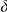.

## Results

**Figure 3:**
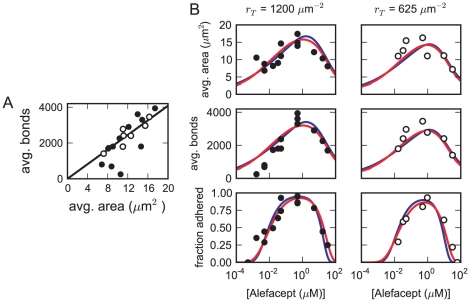
Adhesion data and model fits. A: Experimental data on average bridging bond count versus average contact area. We plot the model with 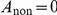 and 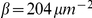 (line). B: Fits to contact area, bridging bond, and fraction-adhered data for the best-fit model with all T-cell epitopes mobile (blue lines) and the best-fit model with epitope immobility 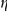 a linear function of the contact area 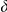 (red lines).

Experiments were previously performed to characterize alefacept-mediated bridging of CD2 epitopes on Jurkat T-cells to fluorescently-labeled Fc
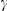
RIIIb receptors on supported bilayers [12]. The alefacept concentration 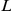 was varied from 0.001 to 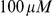, and the fraction of cells bound to the bilayer, the average size of the contact area, and the average number of bonds in the contact area were determined. These types of measurements were made at two different Fc
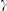
RIIIb densities 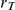 on the bilayer: 1200 and 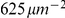. We fit several models of increasing complexity to this data. As seen in Fig. 3A, it was not obvious from our data whether the average contact area goes to zero as the average number of bridging bonds goes to zero, so our fits included possible nonspecific adhesion. Using these models, we then considered the requirements for alefacept-mediated T-cell adhesion, deriving compact expressions for the limiting alefacept concentrations. Finally, we considered the effect of background nonspecific IgG on adhesion.

### Fit of fully mobile model

We first considered an adhesion model with free diffusion of all Fc
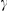
RIIIb receptors on the bilayer and all CD2 epitopes on the T cell. Simultaneous nonlinear least-squares fits of this model to the data are shown by the solid lines in Fig. 3B. We weighted the experiments so that both the bond and contact area data went between zero and one, and we fit six parameters: the bridging bond density 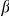, the equilibrium constants 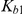 and 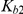, the nonspecific contact area 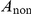, and the Weibull distribution parameters 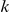 and 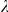. Table 2 lists the best-fit values of the free parameters along with 95% confidence intervals. Note that the best-fit value of the nonspecific contact area 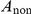 is 0. The best-fit value for the average number of epitopes per cell 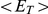 is roughly 5,200. This is much smaller than the average value of 64,000 determined by direct numerical integration of the distribution obtained from flow cytometry (Fig. 2). A reasonable fit could not be obtained when the distribution of epitopes per cell was taken directly from the flow cytometry data, because the model always dramatically overestimated the contact area and number of bridging bonds. Note that previous experiments [12] on Jurkat T cells showed that, on average, each cell could bind up to 20,000 alefacept molecules. This suggests that at least 20,000 and up to 40,000 CD2 epitopes were available for alefacept binding; both values are substantially larger than the best-fit 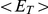 from this model.

**Table 2:** Fit results (best-fit values and 95% confidence intervals).

model	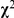	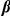 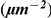	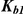 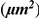	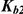 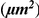	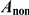 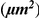	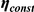 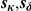	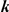	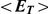 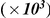
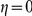	1.190	204	0.6	41	0		1.5	5.2
		176–222	0.2–1.6	22–91	0–0.6		.4–1.7	3.8–6.7
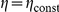	1.190	204	0.6	43	0	0	1.5	5.2
		180–217	0.3–1.3	23–87	0–0.04	0–0.18	1.4–1.7	4.2–6.9
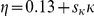	1.203	201	0.5	36	0	0	1.6	6.1
		179–218	0.4–1.3	22–82	0–0.05	0–0.04	1.4–1.8	4.6–7.1
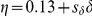	0.961	203	0.4	4.2	0	23	1.2	17
		176–224	0.2–0.7	1.8–13.1	0–0.8	15–28	1.1–1.4	10–27

One counter-intuitive property of the fits is that at high and low ligand concentrations it appears as if there is a slow decline in the average contact area and number of bridging bonds long after the number of bound cells has gone to zero. In these cases, the average contact area is being calculated over the miniscule fraction of cells that are adhered. For example, with 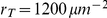, at 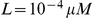, an average of 125 bonds per adhered cell is predicted, but this involves only a fraction 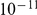 of the total cells. In the experiments, only a few hundred cells were sampled per data point, and the tails of the Weibull distribution may be a poor description of the cell population. Whether the Weibull distribution is a reasonable description of the epitope density on a target cell population *in vivo* is an open question, so we have also considered a lognormal distribution. Although it can fit the flow cytometry data in Fig. 2 well, when it is used to analyze the data in Fig. 3, it predicts that as the ligand concentration decreases, the average contact area and number of bridging bonds go through minima and then rise, yielding a very poor fit to this data (Figure S3). Thus, for the lognormal distribution (and possibly other distributions) the average number of bonds in the contact region can increase as the ligand concentration goes to zero.

This model with freely diffusing CD2 epitopes dramatically underestimates the amount of CD2 present on the T cells. Therefore we considered more complex models incorporating immobile CD2 epitopes in the following section.

### Fit of models with epitope immobility

Prior experiments on Jurkat T cells found that 13% of CD2 epitopes were immobile in the absence of ligand [12]. Furthermore, cell stimulation in T cells may increase CD2 immobility [34]. Thus we extended our mathematical model to include potential CD2 epitope immobility and fit several such models to the data.

We first considered a model in which the immobile epitope fraction 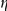 was a fit constant. In this case, the best-fit value of 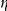 was found to be zero, yielding an identical fit to the fully mobile case. This motivated us to consider models in which the immobile fraction was a function of the fraction 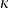 of epitopes cross-linked by ligand or the fractional area 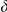 of the specific contact region.

When we fit a model in which the immobile fraction was a linear function of 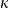 (restricting our search to slopes 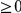), the best-fit value for the slope of that function was 0, yielding a constant immobile fraction of 0.13. The resulting model fit was slightly worse than the completely mobile model.

The red curves in Fig. 3B show the results from a fit with the immobile fraction a linear function of the specific contact area 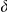. In this case, the fit is somewhat improved, and the best-fit estimate of total epitope count per cell is driven upward to roughly 17,000. This estimate of 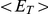 is still only about a third of the value inferred from the flow cytometry data, but it is much closer than the other models. The best-fit function for 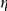 is 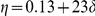, so the immobile fraction increases very rapidly with cell adhesion. Note that in our data, the largest average specific contact area seen is roughly 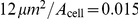 so that only small values of 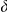 are typically explored, and adding higher-order terms to 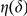 yields negligible improvement in the fit.

### Requirements for adhesion

**Figure 4:**
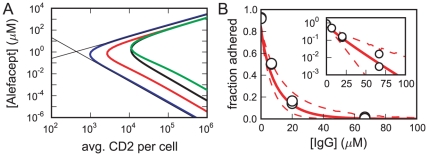
Adhesion model predictions. A: Curves shown enclose the region of greater than 50% cell adhesion for the best-fit all-mobile model (blue), the best-fit model with 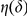 (red), the 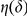 model with 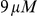 of nonspecific IgG (black), and the 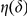 model with ligand-epitope binding constants 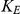 and 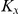 each divided by 10 and no nonspecific IgG (green). The thin solid lines show our approximations for the bounding ligand concentrations 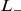 and 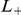. B: Experimental data on the inhibition of adhesion by nonspecific IgG (open circles) compared with predictions from our model with the immobile epitope fraction a function of the contact area. The solid line is from the best-fit model, and the dashed lines denote 95% confidence intervals from our bootstrap parameter uncertainties. Inset plots the same data and prediction on a logarithmic scale.

For drug design, an important consideration is what combinations of ligand concentration and target cell epitope count will yield adhesion. The curves in Fig. 4A separate the region where more than 50% of cells are adhered (inside each curve) from the region where less than 50% are adhered for three different scenarios. The outermost blue curve is the predicted separation curve for the parameters obtained from the fit with all receptors mobile (that shown by the blue lines in Fig. 3B). From this curve we can see that the minimal ligand concentration for adhesion 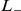 is inversely proportional to the square of the epitope density: the bottom portion of the curve has a slope of approximately negative two.

From the complete set of equations for the model with 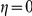, 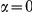, and 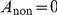, we can obtain simple approximations for 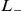 and 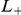 for a fixed target cell epitope density 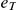:
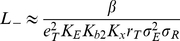
(21)and
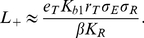
(22)To approximate 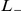, we assumed that, at the lowest ligand concentrations that mediate adhesion, all bridging bonds arise from epitopes bound bivalently (so 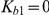). For the 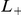 approximation, we assumed that, at the highest ligand concentrations that mediate adhesion, all ligands bound to epitopes are bound singly (so 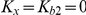). As seen in Fig. 4A, Eq. 21 and 22 closely predict the ligand concentration of 50% adhesion, when we replace 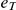 by the average epitope density. Our approximate expression for 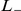 suggests that ligands similar to alefacept can achieve considerable selectivity in epitope density on adhered cells, because 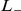 falls with the square of the epitope density. Further, because the ligand-epitope cross-linking constant 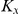 is proportional to the ligand-epitope binding constant 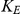, Eq. 21 implies that 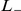 falls inversely with the square of 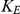, suggesting that a good strategy for lowering 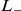 is to develop ligands with higher 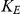 values.

The red curve in Fig. 4A bounds the region of adhesion for the best-fit model in which the immobile fraction 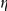 is a linear function of the contact area 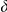. Again, the minimal ligand concentration for adhesion falls as the square of the epitope density. Our expressions for 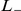 and 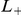 (Eq. 21 and 22) are no longer good approximations in this case, but the dependence of 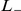 on the ligand-epitope binding constants 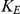 and 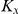 is the same. This is illustrated by the green curve that bounds the region of adhesion for the same model and parameters, with the exception that 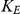 and 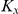 have been divided by 10. As expected from Eq. 21, the minimal ligand concentration for adhesion has increased by a factor of 100.

### Adhesion inhibition by nonspecific IgG

In vivo, alefacept-mediated adhesion depends not only on the concentration of alefacept and the density of CD2 on the T cells, but also on the presence of background nonspecific IgG, which can bind the NK cell receptor and inhibit adhesion. To test the sensitivity of adhesion to background concentrations of nonspecific IgG, the percentage of cells bound to the bilayer was determined in the presence of 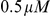 of the ligand (alefacept) and differing concentrations 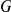 of purified human IgG, with a receptor concentration in the bilayer of 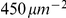 [12]. Nonspecific IgG inhibits cell adhesion by reducing the free receptor concentration (Eq. 14). To have a substantial effect, 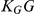 must be greater than 1, where in these experiments the IgG binding constant 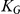 is equal to our 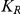. In Fig. 4B, an inhibition of 50% is achieved with an IgG concentration of about 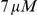, suggesting that 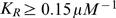, consistent with the value of 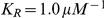 [35]–[37] we took in our fits. Fig. 4B shows good agreement between our predicted inhibition curve using the best-fit parameters for the 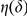 model and the experimental data, providing further validation of our model.

The effect of nonspecific IgG can be interpreted as reducing both the effective total receptor concentration and the effective ligand-receptor binding equilibrium constant. In the equation for the free receptor concentration (Eq. 14), if we replace 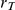 by an effective 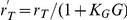 and replace 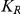 by an effective 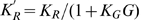, we obtain 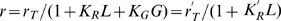. Because the model depends on 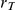 and 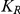 only through 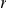, changing variables to an effective 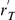 and 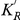 exactly mimics the effect of nonspecific IgG. This allows us to apply our approximations (Eq. 21 and 22) for the minimal 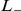 and maximal 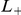 ligand concentrations necessary for substantial adhesion to the case with competing nonspecific IgG. In our expression for 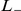 (Eq. 21), 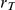 appears only in the denominator, so the effect of adding non-specific IgG to the system is to increase 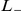 by a factor of 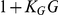. In our expression for 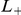 (Eq. 22), 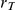 appears in the numerator and 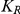 appears in the denominator, so the effects cancel, and 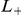 is unchanged. These changes in 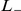 and 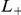 are illustrated by the black curve in Figure 4A, which encloses the region of greater than 50% adhesion when the concentration of IgG is 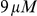, so that 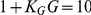, for our best-fit model in which the immobile fraction 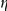 is a linear function of the contact area 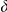.

## Discussion

We have developed an equilibrium model for the ligand-mediated adhesion of cells to surfaces. Our model incorporates immobility of epitopes, potential nonspecific adhesion forces, heterogeneities in target cell epitope density, and the possibility that ligand binding or adhesion alters the immobile epitope fraction. We have applied our model to experiments on the alefacept-mediated adhesion of Jurkat T cells expressing CD2 to bilayer membranes containing the receptor Fc
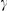
RIIIb, a close relative of the relevant receptor on natural killer cells. We find that our data are best described by a model in which the immobile epitope fraction is a function of the contact area between the target cell and bilayer. Nevertheless, our best-fit model still underestimates the epitope density on Jurkat T cells, perhaps indicating that other factors influence CD2-mediated adhesion and opening a direction for future study.

Our results also suggest general guidelines for the design of immuno-adhesive molecules. We find that for bivalent ligands the minimal ligand concentration 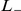 required for adhesion is inversely related to the square of the target cell epitope density, illustrating the potential selectivity of these ligands. We also show that 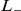 is a quadratic function of the epitope-ligand binding constants, even for our more complex models, suggesting that tuning this interaction may be a fruitful route for drug design.

It is instructive to compare our fit parameters with those from previous investigations of Jurkat T cell adhesion. A previous analysis of adhesion to bilayers containing the natural CD2 binding partner CD58 found a nonspecific contact area of 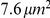 [29]. We, however, find that the nonspecific contact area is driven to zero in all our fits. That previous analysis found the density of bridging bonds 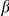 at equilibrium to be approximately 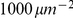, substantially greater than our value of 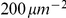. This is unsurprising, because the direct CD2–CD58 interaction draws the cell closer to the bilayer, leading to a larger repulsive force that requires more bonds to overcome. Our values for the 2D association constant 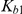 between the alefacept-epitope complex and CD16b are of order 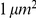. These values are similar to previous results for the 2D association constant between CD2 and CD58, which range from 0.1 to 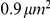 [16], [34], [38]. Overall, our fit parameters are largely consistent with those from the literature.

Our data are best described by a model in which the level of immobilized CD2 on the T cell is proportional to the contact area, rather than the degree of CD2 cross-linking. This suggests that signaling from isolated cross-linked CD2 pairs may be less effective than signaling from larger aggregates or from regions of high CD2 density, such as the contact region. A similar effect is seen in signaling from the immune receptor Fc
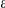
RI on rat basophilic leukemia cells, in which receptor dimers signal weakly compared to larger aggregates [39]. Furthermore, recent experiments have observed signaling in Jurkat T cells adhering to bilayers presenting CD58, the natural binding partner of CD2 [40].

Our models assume immobilization is a global effect, affecting all CD2 epitopes; we make this assumption because localized models of immobility yield equilibrium behavior identical to the fully mobile model, which does not fit our data. For example, a model might assume that only receptors cross-linked by ligand are immobilized. However, because ligand binding (and thus cross-linking) is reversible, any cross-linked pair of receptors will eventually break up and become mobile again. In equilibrium, this model would thus yield identical results to allowing all receptors to be mobile. Similarly, a model might assume that only receptors within the contact area were immobilized, so that all receptors outside the contact area would be free to diffuse to the contact area. Given that immobile receptors have very similar binding properties to mobile receptors once they reach the contact region, such a model should also yield very similar results to the fully mobile model.

In our system, comparing the bridging bond density 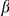 of approximately 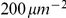 with the initial cellular CD2 density of roughly 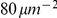, we see that the density increase in the contact region is roughly a factor of 2.5. This increases the fraction of CD2 nearest neighbors within a given distance by the same factor of 2.5 (Eq. 1), similarly increasing the probability of interaction. Thus the increased density of CD2 in the contact region may promote interactions between CD2 molecules and drive signaling. When alefacept adheres T cells to NK cells in vivo, Fc
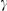
RIIIa receptors on the NK cell will be similarly concentrated in the contact region, and this concentration may contribute to the signal that drives NK-mediated killing of target cells. Moreover, we expect the repulsive force to be greater between cells than between a cell and a bilayer; therefore, the in vivo bridging bond density is probably larger, leading to greater in vivo receptor concentration than seen in our experiments.

In summary, we have developed an equilibrium model for the immunoadhesin-mediated adhesion of cells to surfaces. Our analysis suggests guidelines for the design of therapeutic immunoadhesins. Furthermore, applying our model to experiments on Jurkat T cells suggests that an active cellular process may be increasing CD2 immobility in response to alefacept-mediated surface adhesion.

## Supporting Information

Figure S1
**Sensitivity of our results to** 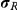 **,** 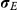 **, and** 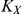 **.** A: As 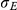 and 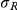 vary from our assumed value of 0.75, the plot shows relative changes of best-fit results for the completely mobile model with 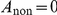. Inset shows the same data on a logarithm scale. Most results are very insensitive, but the best-fit values for 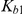 and 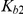 do depend on 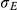 and 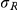. B: As 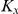 varies from our assumed value, the plot shows relative changes of best-fit results for the completely mobile model with 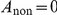. Inset shows the same data on a logarithm scale. Most results are very insensitive, but the best-fit value of 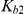 is inversely proportional to the assumed value of 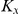.(EPS)

Figure S2
**Illustrative model solution with divergence at large** 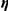 **.** Open circles show the solution for 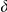 as a function of 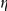 for a particular set of model parameters. The solid black line is the filtered solution, accounting for the divergence and fixing 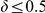. The red line is the function 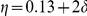, and the resulting solution for the model in which 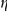 depends on 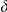 is shown by the red star. The dashed green line is the fraction 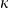 of cross-linked epitopes as a function of 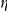, and the solid green line is the function 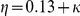. The resulting solution of the model in which 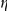 depends on 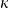 is shown by the green diamond.(EPS)

Figure S3 **Model with lognormal distribution of epitope counts.** Shown is the best-fit model with all epitopes mobile.
(EPS)

Text S1 **Full model details and derivations.**
(PDF)

## References

[1] Iannello A, Ahmad A (2005). Role of antibody-dependent cell-mediated cytotoxicity in the efficacy of therapeutic anti-cancer monoclonal antibodies.. Cancer Metastasis Rev.

[2] da Silva AJ, Brickelmaier M, Majeau GR, Li Z, Su L (2002). Alefacept, an immunomodulatory recombinant LFA-3/IgG1 fusion protein, induces CD16 signaling and CD2/CD16-dependent apoptosis of CD2+ cells.. J Immunol.

[3] Trapani JA, Smyth MJ (2002). Functional significance of the perforin/granzyme cell death pathway.. Nat Rev Immunol.

[4] Bryceson YT, March ME, Barber DF, Ljunggren HG, Long EO (2005). Cytolytic granule polarization and degranulation controlled by different receptors in resting NK cells.. J Exp Med.

[5] Ghetie MA, Podar EM, Ilgen A, Gordon BE, Uhr JW (1997). Homodimerization of tumor-reactive monoclonal antibodies markedly increases their ability to induce growth arrest or apoptosis of tumor cells.. Proc Nat Acad Sci USA.

[6] Friedman LM, Rinon A, Schechter B, Lyass L, Lavi S (2005). Synergistic down-regulation of receptor tyrosine kinases by combinations of mAbs: Implications for cancer immunotherapy.. Proc Nat Acad Sci USA.

[7] Clynes RA, Towers TL, Presta LG, Ravetch JV (2000). Inhibitory Fc receptors modulate in vivo cytoxicity against tumor targets.. Nat Med.

[8] Waldmann TA (2003). Immunotherapy: Past, present and future.. Nat Med.

[9] Arnould L, Gelly M, Penault-Llorca F, Benoit L, Bonnetain F (2006). Trastuzumab-based treatment of HER2-positive breast cancer: an antibody-dependent cellular cytotoxicity mechanism?. Br J Cancer.

[10] Koon H, Severy P, Hagg D, Butler K, Hill T (2006). Antileukemic effect of daclizumab in CD25 high-expressing leukemias and impact of tumor burden on antibody dosing.. Leuk Res.

[11] Preithner S, Elm S, Lippold S, Locher M, Wolf A (2006). High concentrations of therapeutic IgG1 antibodies are needed to compensate for inhibition of antibody-dependent cellular cytotoxicity by excess endogenous immunoglobulin G.. Mol Immunol.

[12] Dustin ML, Starr T, Coombs D, Majeau GR, Meier W (2007). Quantification and modeling of tripartite CD2-, CD58FC chimera (alefacept)-, and CD16-mediated cell adhesion.. J Biol Chem.

[13] Scallon BJ, Scigliano E, Freedman VH, Miedel MC, Pan YC (1989). A human immunoglobulin G receptor exists in both polypeptide-anchored and phosphatidylinositol-glycan-anchored forms.. Proc Natl Acad Sci USA.

[14] Anderson C, Looney R, Culp D, Ryan D, Fleit H (1990). Alveolar and peritoneal macrophages bear three distinct classes of Fc receptors for IgG.. J Immunol.

[15] Dustin ML, Springer TA (1991). Role of lymphocyte adhesion receptors in transient interactions and cell locomotion.. Annu Rev Immunol.

[16] Dustin ML, Golan DE, Zhu DM, Miller JM, Meier W (1997). Low affinity interaction of human or rat T cell adhesion molecule CD2 with its ligand aligns adhering membranes to achieve high physiological affinity.. J Biol Chem.

[17] Majeau G, Meier W, Jimmo B, Kioussis D, Hochman P (1994). Mechanism of lymphocyte function-associated molecule 3-Ig fusion proteins inhibition of t cell responses. structure/function analysis in vitro and in human CD2 transgenic mice.. J Immunol.

[18] Ellis CN, Krueger GG (2001). Treatment of chronic plaque psoriasis by selective targeting of memory effector T lymphocytes.. N Engl J Med.

[19] Cooper J, Morgan G, Harding S, Subramanyam M, Majeau G (2003). Alefacept selectively promotes NK cell-mediated deletion of CD45R0^+^ human T cells.. Eur J Immunol.

[20] Chamian F, Lowes MA, Lin SL, Lee E, Kikuchi T (2005). Alefacept reduces infiltrating T cells, activated dendritic cells, and inflammatory genes in psoriasis vulgaris.. Proc Natl Acad Sci USA.

[21] Byrn RA, Mordenti J, Lucas C, Smith D, Marsters SA (1990). Biological properties of a CD4 immunoadhesin.. Nature.

[22] Ashkenazi A, Chamow SM (1995). Immunoadhesins: An alternative to human monoclonal antibodies.. Methods.

[23] Dustin M, Ferguson L, Chan P, Springer T, Golan D (1996). Visualization of CD2 interaction with LFA-3 and determination of the two-dimensional dissociation constant for adhesion receptors in a contact area.. J Cell Biol.

[24] Chandrasekhar S (1943). Stochastic problems in physics and astronomy.. Rev Mod Phys.

[25] Harris LJ, Larson SB, Hasel KW, Day J, Greenwood A (1992). The three-dimensional structure of an intact monoclonal antibody for canine lymphoma.. Nature.

[26] Wofsy C, Goldstein B (1992). Interpretation of Scatchard plots for aggregating receptor systems.. Math Biosci.

[27] Bell G, Dembo M, Bongrand P (1984). Cell adhesion. Competition between nonspecific repulsion and specific bonding.. Biophys J.

[28] Rosenbluth MJ, Lam WA, Fletcher DA (2006). Force microscopy of nonadherent cells: A comparison of leukemia cell deformability.. Biophys J.

[29] Shao JY, Yu Y, Dustin ML (2005). A model for CD2/CD58-mediated adhesion strengthening.. Ann Biomed Eng.

[30] Bell GI (1981). Estimate of the sticking probability for cells in uniform shear flow with adhesion caused by specific bonds.. Cell Biochem Biophys.

[31] Hammer DA, Lauffenburger DA (1989). A dynamical model for receptor-mediated cell adhesion to surfaces in viscous shear flow.. Cell Biochem Biophys.

[32] Jones E, Oliphant T, Peterson P (2001–). SciPy: Open source scientific tools for Python.. http://www.scipy.org/.

[33] Wells AF, Miller CE, Nadel MK (1966). Rapid fluorescein and protein assay method for fluorescent-antibody conjugates.. Appl Microbiol.

[34] Zhu DM, Dustin ML, Cairo CW, Thatte HS, Golan DE (2006). Mechanisms of cellular avidity regulation in CD2–CD58-mediated T cell adhesion.. ACS Chem Biol.

[35] Galon J, Robertson MW, Galinha A, Maziéres N, Spagnoli R (1997). Affinity of the interaction between Fc gamma receptor type III (Fc*γ*RIII) and monomeric human IgG subclasses. role of Fc*γ*III glycosylation.. Eur J Immunol.

[36] Chesla SE, Li P, Nagarajan S, Selvaraj P, Zhu C (2000). The membrane anchor influences ligand binding two-dimensional kinetic rates and three-dimensional affinity of Fc*γ*RIII (CD16).. J Biol Chem.

[37] Maenaka K, van der Merwe PA, Stuart DI, Jones EY, Sondermann P (2001). The human low affinity Fc*γ* receptors IIa, IIb, and III bind IgG with fast kinetics and distinct thermodynamic properties.. J Biol Chem.

[38] Zhu DM, Dustin ML, Cairo CW, Golan DE (2007). Analysis of two-dimensional dissociation constant of laterally mobile cell adhesion molecules.. Biophys J.

[39] Fewtrell C, Metzger H (1980). Larger oligomers of IgE are more effective than dimers in stimulating rat basophilic leukemia cells.. J Immunol.

[40] Kaizuka Y, Douglass AD, Vardhana S, Dustin ML, Vale RD (2009). The coreceptor CD2 uses plasma membrane microdomains to transduce signals in T cells.. J Cell Biol.

