# Supplementary material for: A Biophysical Model of Cell Adhesion Mediated by Immunoadhesin Drugs and Antibodies
Source: PLoS One. 2011 May 18;6(5):e19701. doi: 10.1371/journal.pone.0019701 (PMC3100730; doi:10.1371/journal.pone.0019701)
Supplement: Text S1 — Full model details and derivations. (PDF) [file pone.0019701.s004.pdf]

Text S1: A biophysical model of cell adhesion mediated by immunoadhesin drugs and antibodies

Full model

The full set of equilibrium relations among the molecular complexes considered in our model is given by Eq. S1 through S18, which express all complex concentrations as functions of e, r, i, and i_{in}:

$$e_{\text{in}} = \sigma_E e, \tag{S1}$$
$$r_{\text{in}} = \sigma_R r, \tag{S2}$$
$$e_1 = 2K_E L e, \tag{S3}$$
$$e_2 = K_x e_1 e/2, \tag{S4}$$
$$e_{1\text{in}} = 2K_E L e_{\text{in}}, \tag{S5}$$
$$e_{2\text{in}} = K_x e_{1\text{in}} e_{\text{in}}/2, \tag{S6}$$
$$b_{10} = K_{b10} e_{1\text{in}} r_{\text{in}}, \tag{S7}$$
$$b_{20} = K_{b20} e_{2\text{in}} r_{\text{in}}, \tag{S8}$$
$$r_1 = K_R L r, \tag{S9}$$
$$r_{1\text{in}} = K_R L r_{\text{in}}, \tag{S10}$$
$$i_1 = 2K_I L i, \tag{S11}$$
$$i_{1\text{in}} = 2K_I L i_{\text{in}}, \tag{S12}$$
$$h = K_{HE} e_1 i, \tag{S13}$$
$$h_{\text{in}} = K_{HE} e_{1\text{in}} i_{\text{in}}, \tag{S14}$$
$$b_{01} = K_{b01} i_{1\text{in}} r_{\text{in}}, \tag{S15}$$
$$b_{11} = K_{FH} h_{\text{in}} r_{\text{in}}, \tag{S16}$$
$$r_G = K_G G r, \text{ and} \tag{S17}$$
$$r_{G\text{in}} = K_G G r_{\text{in}}. \tag{S18}$$

Note that the above equations do not involve any of the underscored equilibrium constants indicated in Fig. 1. This is because enforcing detailed balance around all loops in the reaction diagram introduces six constraints on the equilibrium constants, which we use to eliminate the underlined constants:

$$K_x K_{b20} = \underline{K_x} K_{b10}, \tag{S19}$$
$$K_E K_{b10} = \underline{K_E} K_R, \tag{S20}$$
$$K_{b10} \underline{K_{FE}} = K_{FH} K_{HE}, \tag{S21}$$
$$K_I K_{b01} = \underline{K_I} K_R, \tag{S22}$$
$$K_E K_{HE} = K_I \underline{K_{HI}}, \text{ and} \tag{S23}$$
$$K_I K_{b01} \underline{K_{FI}} = K_{FH} K_{HE} K_E. \tag{S24}$$

Requirements for adhesion

For the model with all receptors mobile ($\eta = 0$), no receptor depletion on the bilayer ($\alpha = 0$), and no non-specific adhesion ($A_{\text{non}} = 0$), we can find equations for the bounds on ligand concentration L and epitope density e_T for adhesion by setting $\delta = 0$ in Eq. 6, 13, and 16. We can also avoid direct integration when calculating the average adhered area.

As described in the main text, to find the minimal epitope density $e_{T\text{min}}$ for adhesion, we plug $\delta = 0$ into our model equations. For the case of $\alpha = \eta = A_{\text{non}} = 0$, plugging in our expressions for equilibrium species concentrations yields a system of three equations for the unknowns e, r, and L. For fixed L, these three equations can be reduced to a single quadratic equation:

$$a_e\, e_T^2 + b_e\, e_T + c_e = 0, \tag{S25}$$

where the coefficients are given by:

$$\begin{aligned}
a_e &= {K'_{b2}}^2 K_E K_x L r_T^2, && \text{(S26)}\\
b_e &= 2K_E L r_T \big[-4{K'_{b1}}^2 K_E L\, r_T + K'_{b1}K'_{b2} r_T (1 + 2K_E L) \\
&\qquad - 2K'_{b2}K_x\beta(1 + K_R L + K_G G)\big], \text{ and} && \text{(S27)}\\
c_e &= \beta(1 + K_R L + K_G G)\big\{4K_E L\big[K'_{b1}(r_T + 2K_E L r_T) \\
&\qquad + \beta K_x(1 + K_R L + K_G G)\big] - K'_{b2} r_T (1 + 2K_E L)^2\big\}, && \text{(S28)}
\end{aligned}$$

with $K'_{b1} \equiv \sigma_R \sigma_E K_{b1}$ and $K'_{b2} \equiv \sigma_R \sigma_E^2 K_{b2}$. Eq. S25 has two solutions, the larger of which is $e_{T\text{min}}$. For fixed epitope concentration e_T, the system reduces to a single cubic equation:

$$a_L\, L^3 + b_L\, L^2 + c_L\, L + d_L = 0, \tag{S29}$$

where the coefficients are given by:

$$\begin{aligned}
a_L &= 4\beta K_E K_R (2r_T K'_{b1} K_E - r_T K'_{b2} K_E + \beta K_R K_x), && \text{(S30)}\\
b_L &= -4K_E\big\{\beta r_T\big[K'_{b2}(K_E + K_R) - K'_{b1} r_T (2K_E + K_R) \\
&\quad - 2K_R K_x \beta\big] + e_T r_T (2{K'_{b1}}^2 K_E r_T \\
&\qquad - K'_{b1}K'_{b2}K_E r_T + K'_{b2}K_R K_x \beta)\big\}, && \text{(S31)}\\
c_L &= 2r_T K'_{b1} K_E (2\beta + e_T r_T K'_{b2}) + 4\beta^2 K_E K_x \\
&\qquad + e_T^2 r_T^2 {K'_{b2}}^2 K_E K_x \\
&\qquad - r_T \beta K'_{b2}\left[K_R + 4K_E(1 + K_x e_T)\right], \text{ and} && \text{(S32)}\\
d_L &= -r_T \beta K'_{b2}. && \text{(S33)}
\end{aligned}$$

Eq. S29 has either two positive solutions, which are L_- and L_+, or no positive solutions, in which case adhesion is not possible for any ligand concentration.

When $\alpha = \eta = A_{\text{non}} = 0$, then we do not need to explicitly integrate over $f(e_T)$, because

$$\langle \delta \rangle = \delta(\langle e_T \rangle), \tag{S34}$$

where $\langle e_T \rangle$ is the average epitope density of adhered cells:

$$\langle e_T \rangle = \frac{\int_{e_{T\text{min}}}^{\infty} e_T f(e_T)\, de_T}{\int_{e_{T\text{min}}}^{\infty} f(e_T)\, de_T}. \tag{S35}$$

To prove this, we first note the constraint imposed by our relation for the bond density (Eq.16) when $\alpha = \eta = A_{\text{non}} = 0$. In this case, after substituting our equilibrium relations, β is equal to a function of e and

r which does not involve e_T. When $\alpha = 0$, r is simply $r_T/(1 + K_R L)$ (from Eq. 13), again not involving e_T. Thus, our relation for the bond density provides a constraint on the free epitope density e that is independent of the total epitope density e_T. In other words, all adhered cells will have the same density of free epitopes and thus of all other complexes, irrespective of their total epitope density e_T.

When $\alpha = \eta = 0$, our conservation equation for mobile epitopes (Eq. 6) yields the following expression for δ:

$$\delta = \frac{e_T - (e + e_1 + 2e_2)}{b_{10} + 2b_{20} + e_{\text{in}} + e_{1\text{in}} + 2e_{2\text{in}} - (e + e_1 + 2e_2)}. \tag{S36}$$

Note that all complex concentrations on the right-hand side (e, e_1, e_2, b_{10}, b_{20}, e_{in}, $e_{1\text{in}}$, and $e_{2\text{in}}$) are functions of e and r only, which we have just shown are independent of e_T. Thus, taking the average over adhered cells, we have

$$\langle\delta\rangle = \frac{\langle e_T\rangle - (e + e_1 + 2e_2)}{b_{10} + 2b_{20} + e_{\text{in}} + e_{1\text{in}} + 2e_{2\text{in}} - (e + e_1 + 2e_2)}, \tag{S37}$$

where the right-hand side is simply the specific contact area calculated using the average epitope density of adhered cells $\langle e_T\rangle$. Thus, to calculate $\langle\delta\rangle$ we need only calculate $e_{T\text{min}}$, then $\langle e_T\rangle$, and finally $\langle\delta\rangle$.
